# Hong-Bai-Lan-Shen Extract Alleviates the CoCl_2_-Induced Apoptosis in H9C2 Cells by Regulating the AMPK Pathway

**DOI:** 10.3390/vetsci12030267

**Published:** 2025-03-13

**Authors:** Jinxue Ding, Jinwu Meng, Wenjia Wang, Bolin Gu, Mengxin Hu, Jiaguo Liu

**Affiliations:** 1 College of Animal Science, Anhui Science and Technology University, Chuzhou 233100, China; dingjx@stu.njau.edu.cn; 2 Institute of Traditional Chinese Veterinary Medicine, College of Veterinary Medicine, Nanjing Agricultural University, Nanjing 210095, China; 2020207033@stu.njau.edu.cn (J.M.); 2019207050@njau.edu.cn (W.W.); 2022107089@stu.njau.edu.cn (B.G.); 2022207016@stu.njau.edu.cn (M.H.); 3 College of Agriculture, Jinhua University of Vocational Technology, Jinhua 321000, China; 4 School of Animal Science, Ningxia University, Yinchuan 750002, China

**Keywords:** LC-MS/MS, Hong-bai-lan-shen extract, H9C2 cell, CoCl_2_, cell apoptosis, AMPKα

## Abstract

This study aims to explore the protective effects of Hong-bai-lan-shen (HBLS) extract, a traditional Chinese medicine compound, on myocardial injury based on metabolomics. H9C2 cells were cultured with HBLS extract for 12 h, and then the cells were cultured in a CoCl_2_-containing medium, a model simulating the ischemic-hypoxic damage in myocardial cells, for an additional 12 h. The cell viability, cytotoxicity, intracellular metabolite and reactive oxygen species (ROS), mitochondrial membrane potential, apoptosis, and adenosine monophosphate-activated protein kinase (AMPK) signal pathway were determined. The results showed that HBLS extract significantly increased cell viability, stabilized cell morphology, reduced lactate dehydrogenase (LDH) release and ROS production, blocked cysteine-aspartic acid protease 3 (caspase-3) and bcl-2-associated X protein (Bax) expression and decreased apoptotic cell numbers. Meanwhile, HBLS increased membrane potential and the expression of B-cell lymphoma-2 (Bcl-2). Additionally, HBLS extract upregulated the expression of AMPK, PI3K, and protein kinase B (AKT) (*p* < 0.05, *p* < 0.01). These findings suggest that HBLS extract has a protective effect on myocardial cells by regulating the AMPK signal pathway and may be a promising therapeutic candidate for ischemic heart disease.

## Simple Summary

In recent years, there has been an increasing number of animal heart diseases caused by various reasons. Heart diseases caused by hypoxia have seriously affected the quality of life of animals. Traditional Chinese medicines such as Rhodiola rosea have shown good efficacy in the treatment of ischemic heart diseases. In this study, based on the principles of traditional Chinese medicine, a scientific formula was developed. Guided by the results of metabolomics, the protective mechanism of Hong-bai-lan-shen extract on H9C2 myocardial cells was investigated in depth. This comprehensive research method is expected to provide a clearer and more reliable theoretical basis for the research and development of new drugs. At the same time, it also helps to gain a deeper understanding of the mechanism of action of traditional Chinese medicine in animal heart health.

## 1. Introduction

Prolonged exposure of the myocardium to ischemic and hypoxic conditions can lead to pathological cardiac hypertrophy and irreversible morphological changes [1,2]. Heart disease not only poses a threat to human health but also affects the lives and well-being of animals. For instance, activities such as chasing and searching tasks performed by police dogs or endurance racing events involving horses require high-intensity exercise, demanding substantial physical and cardiopulmonary endurance. Prolonged exposure to high-stress levels may increase the risk of heart disease. Therefore, it is crucial to explore safe and effective drugs that can mitigate myocardial damage, benefiting both humans and animals.

Traditional Chinese medicine is a major branch of medicine that utilizes herbal medicine sourced from plants. HBLS, consisting of *Rhodiola rosea* L., *Atractylodes macrocephala* Koidz., *Geum aleppicum* Jacq., and *Codonopsis pilosula* (Franch.) Nannf., follows the principles of traditional Chinese medicine [3]. Studies have shown that these herbs have protective effects on the myocardium and is widely recognized for its safety [4,5,6]. However, the underlying mechanisms of the protective effects of HBLS extract on the myocardium are still not fully elucidated. To better understand the cardioprotective effects of HBLS, a chemical hypoxia model using CoCl_2_ was chosen to simulate the ischemic–hypoxic damage in myocardial cells, this model has been widely used by researchers to investigate the protective mechanisms of new drugs against cardiac injuries, such as α-mangosteen [7], 10-Gingerol [8], and Naringin [9]. In order to gain a comprehensive understanding of the cardioprotective mechanisms of HBLS, metabolomics, an emerging omics technology following genomics, transcriptomics, and proteomics, has also been introduced [10].

## 2. Materials and Methods

Preparation of HBLS extract. The herbs for HBLS were purchased from Tong Ren Tang pharmacy (Nanjing, Jiangsu). The composition of HBLS listed in Table 1 is based on Veterinary Pharmacopeia of People’s Republic of China (version 2010) and Traditional Chinese Veterinary Medicine [11]. The plant name has been checked by http://www.worldfloraonline.org, accessed on 22 January 2024.

In order to explore the correlation between different extraction processes and therapeutic effects, two research systems have been established, namely the aqueous extraction process and the ethanol extraction process. The aqueous extraction process adopts a two-stage water decoction method. This method not only maximizes the retention of components such as polysaccharides and alkaloids but also features a simple extraction process. In contrast, the ethanol extraction process is complex. Through three rounds of ethanol reflux extraction, flavonoid compounds are retained. The specific preparation steps are as follows:

Preparation of the HBLS aqueous extract (HBLS-L) was carried out as follows: 100 g of Chinese herbal compound were soaked in 2 L of water for 30 min. The mixture was then brought to a boil over high heat and simmered for 60 min. The first filtration was performed using three layers of gauze to collect the first filtrate. The residue was boiled for an additional 60 min in 2 L of water, and the second filtrate was collected. The two filtrates were combined and filtered again using three layers of gauze. The resulting solution was centrifuged at 5000 r/min for 10 min at 4 °C, and the supernatant was collected. The concentrated extract was obtained using a rotary evaporator at a temperature of 65 °C, resulting in a final volume of 100 mL [16].

Preparation of HBLS ethanol extract (HBLS-C): 100 g of Chinese herbal compound were soaked in 2 L of 95% ethanol in an evaporating flask for 30 min, followed by boiling for 60 min using a rotary evaporator. The filtrate was collected after passing through three layers of gauze. The residue was further extracted by boiling in an additional 2 L of 95% ethanol for 60 min, and another round of filtration was performed. This process was repeated one more time with 2 L of 95% ethanol. The three filtrates were then combined, filtered, and concentrated to 100 mL using a rotary evaporator [17].

Cell Culture and treatment. H9C2 cells (American type culture collection, Manassas, VA, USA, CRL-1446), at passage 6, were cultivated in high-glucose DMEM supplemented with 10% fetal bovine serum (FBS) and 1% streptomycin/penicillin. The cells were incubated at a temperature of 37 °C in a 5% CO_2_ atmosphere [18].

CoCl_2_ (Sigma-Aldrich, St. Louis, MO, USA, 232696, with a purity of 97%) solution was added to the cell culture medium to obtain different concentrations of CoCl_2_ (100, 200, 300, 400, 500, 600, 700, and 800 μM). H9C2 cells were cultured in the medium containing CoCl_2_ for 12 h. Cell viability was measured using the CCK8 assay and quantified by measuring the absorbance at 450 nm. The IC50 value was calculated using Prism 8.4.2 software to determine the optimal dosage of CoCl_2_. H9C2 cells were co-cultured with varying concentrations of HBLS-L and HBLS-C in the cell culture medium for 12 h. Subsequently, the medium was replaced with a CoCl_2_-supplemented medium, and the cells were further cultured for an additional 12 h. Cell viability was determined using the CCK8 assay, and quantitative measurements were obtained at OD450 nm. The appropriate concentrations of HBLS-L and HBLS-C were selected based on the results.

LDH Activity Assay. According to the manufacturer’s instructions, an LDH cell cytotoxicity assay kit was used to determine the LDH released from damaged cells in the supernatant. LDH analysis involved adding 10 μL of culture medium to a 96-well plate. The released LDH caused an increase in absorbance at 450 nm as measured by the spectrophotometer.

Intracellular Metabolite Extraction. The cell culture medium was discarded, and the cells were washed three times with pre-chilled PBS. After removing the supernatant, 1 mL of pre-chilled 80% methanol (HPLC grade) aqueous solution was added. The cells were scraped in the same direction and transferred to a pre-chilled cryotube. The cryotube was then flash-frozen in liquid nitrogen for 5 min and thawed on ice for 30 s. Subsequently, it was sonicated for 6 min and centrifuged at 5000 r/min and 4 °C for 1 min. The supernatant was transferred to a new centrifuge tube and freeze-dried to obtain a dry powder. The corresponding 10% methanol solution was added to dissolve the dried powder, according to the volume of the sample taken. The sample was then subjected to analysis using LC-MS/MS [19]. An equal volume of each experimental sample was taken and mixed as a quality control (QC) sample. The blank sample was prepared by substituting 53% methanol-water solution for the experimental sample, and the pretreatment process was the same as that of the experimental samples.

LC-MS/MS Conditions. Metabolite analysis was performed using a Vanquish UHPLC system coupled with a Q Exactive™ HF-X mass spectrometer (Thermo Fisher Scientific, Waltham, MA, USA). The samples were separated on a Hypesil Gold column (C18) with a flow rate of 0.2 mL/min and a column temperature of 40 °C. In positive mode, the mobile phases A and B were 0.1% formic acid and methanol, respectively. In negative mode, the mobile phases A and B were 5 mM ammonium acetate, pH 9.0, and methanol. The gradient elution program was as follows: 0–1.5 min, 98% A; 1.5–3 min, 15% A; 3–10 min, 0% A; 10–10.1 min, 98% A; 10.1–11 min, 98% A; 11–12 min, 98% A. The mass spectrometer was operated in scanning mode within a range of 100–1500 m/z. The ESI source settings were as follows: spray voltage, 3.5 kV; sheath gas flow rate, 35 psi; auxiliary gas flow rate, 10 L/min; ion transfer tube temperature, 320 °C; ion funnel radio frequency level, 60; auxiliary gas heater temperature, 350 °C. Both positive and negative polarities were employed, and MS/MS analysis was performed in data-dependent mode.

Data Processing. The raw data were imported into the CD3.1 search software for processing. Simple screening of each metabolite was conducted based on parameters such as retention time and mass-to-charge ratio. Subsequently, peak alignment was performed for different samples using a retention time deviation of 0.2 min and a mass deviation of 5 mg/kg, which improved the accuracy of identification. Peak extraction was then carried out using a mass deviation of 5 mg/kg, a signal intensity deviation of 30%, a signal-to-noise ratio of 3, a minimum signal intensity, and summed ions. Quantification of peak areas was conducted, followed by integration of target ions. Molecular formula prediction was achieved by comparing the molecular ion peaks and fragment ions with the mzCloud, mzVault, and Masslist databases (https://www.mzcloud.org/, accessed on 20 January 2024). Background ions were subtracted using blank samples, and the original quantification results were standardized. Ultimately, the identification and relative quantification of metabolites were obtained. Data processing was conducted using the Linux operating system (CentOS version 6.6) and the software tools R 4.4 and Python 3.12.6. The identified metabolites were annotated using the KEGG database (https://www.genome.jp/kegg/pathway.html, accessed on 20 January 2024).

Measurement of Intracellular ROS Generation. DCFH-DA was used to measure intracellular reactive oxygen species levels. Then, 1 mL of DCFH-DA probe (10 μM) was added to the cells in each treated group. The cells were incubated at 37 °C for 30 min with intermittent mixing every 5 min to ensure thorough probe-cell contact. After washing with PBS 3 times, the fluorescence signal of DCF (the oxidized product of DCFH-DA) was immediately observed using the Invitrogen EVOS FL automated imaging system, and the fluorescence intensity was calculated using ImageJ 1.54i software.

Measurement of Cell Apoptosis. After cell processing, cells were digested with pancreatin without EDTA. The mixture was centrifuged at 1800 r/min and 4 °C for 5 min, and the supernatant was discarded. Cells were washed twice with pre-chilled PBS, with each wash performed at 1800 r/min and 4 °C for 5 min. After discarding the supernatant, 100 μL of Binding Buffer was added and gently pipetted to obtain a single-cell suspension. Addition of 5 μL of Annexin V-FITC and 5 μL of PI (Propidium Iodide) followed, with gentle pipetting to ensure homogeneity. The mixture was then incubated at room temperature and protected from light for 10 min. Finally, 400 μL of Binding Buffer was added and gently mixed, and the stained samples were immediately analyzed using a flow cytometer (Beckman Coulter, Inc., Brea, CA, USA), the number of cells aspirated each time is set to one million.

Measurement of Mitochondrial Membrane Potential in Cells. H9C2 cells were digested with trypsin and then terminated with a culture medium. After centrifugation, the cells were washed twice with PBS. Subsequently, 0.5 mL of cell culture medium and 0.5 mL of JC-1 staining working solution were added. The mixture was thoroughly mixed and incubated at 37 °C in a cell culture incubator for 20 min. After incubation, the cells were centrifuged at 600 g for 3 min at 4 °C. The supernatant was discarded, and the pellet was washed twice with JC-1 staining buffer. Then, 300 μL of JC-1 staining buffer was added to resuspend the pellet, followed by analysis using flow cytometry, the number of cells aspirated each time is set to one million.

Western blot Analysis. Cells were lysed and collected to measure the levels of relevant proteins using a RIPA lysis buffer containing the proteinase inhibitor PMSF. The protein concentration of the samples was determined using the BCA concentration assay kit. Protein immunoblot analysis was performed using primary antibodies targeting Caspase-3, Bcl-2, Bax, PI3K, AKT, and AMPKα, followed by secondary antibodies for radiographic visualization. After washing with TBST, the bands were detected using a highly sensitive chemiluminescent gel imaging system, and the grayscale values were quantitatively analyzed using ImageJ software. The ratio of grayscale values between the target protein and the reference protein was used as a relative expression level of the target protein.

Data Analysis. Statistical analysis and visualization were performed using SPSS Statistics 26 and GraphPad Prism 9 software. One-way analysis of variance (ANOVA) and post hoc multiple comparisons using the Least Significant Difference (LSD) test were employed to assess the differences between groups. Mean values, along with their standard deviations (mean ± SD), were presented for the data. Statistical significance was considered at *p* < 0.05.

## 3. Results

### 3.1. HBLS Extract Altered the Cell Viability

**Figure 1:**
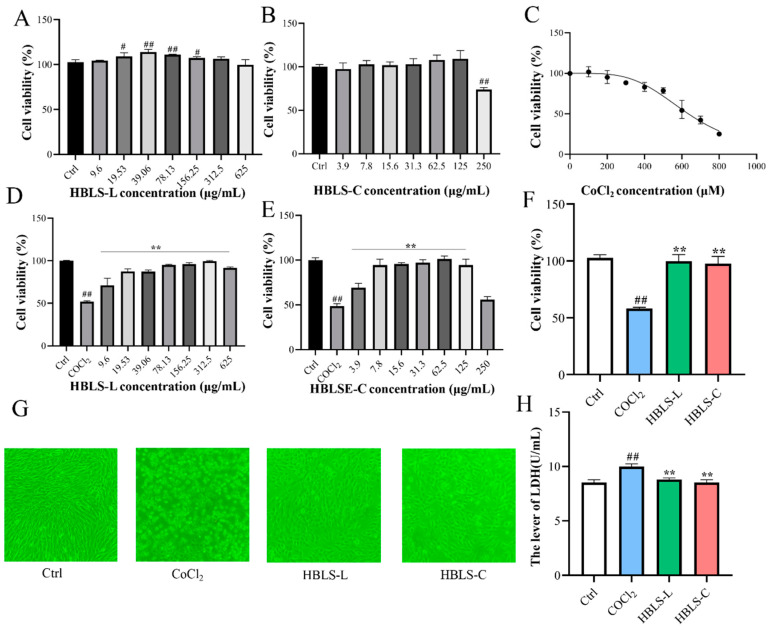
The effect of different concentrations of HBLS extract on the viability of H9C2 cells. (**A**) The effect of HBLS-L on cell viability. (**B**) The effect of HBLS-C on cell viability. (**C**) The effect of CoCl_2_ on cell viability. (**D**) The alleviation of the effect of CoCl_2_ on cell viability by HBLS-L. (**E**) The alleviation of the effect of CoCl_2_ on cell viability by HBLS-C. (**F**) The alleviation of the effect of CoCl_2_ on cell viability by both HBLS-L and HBLS-C. (**G**) Direct observation of changes in cell morphology using an inverted microscope. (**H**) Release of LDH. Data are presented as mean ± SD, indicated as ^#^ *p* < 0.05 and ^##^ *p* < 0.01 compared to the Control group, and ** *p* < 0.01 compared to the CoCl_2_ group, respectively.

The results of the CCK-8 assay showed that HBLS-L concentrations ranging from 9.6 to 625 μg/mL had no negative effect on H9C2 cell viability (Figure 1A). Similarly, HBLS-C concentrations ranging from 3.9 to 125 μg/mL had no negative effect on H9C2 cell viability (Figure 1B). Addition of different concentrations of CoCl_2_ (100, 200, 300, 400, 500, 600, 700, and 800 μM) to H9C2 cells resulted in a dose-dependent decrease in cell viability. The IC50 value for CoCl_2_ concentration was determined to be 634.8 μM (Figure 1C). For the convenience of experimental drug preparation, a CoCl_2_ concentration of 650μM was chosen, which resulted in a cell viability of 52.26 ± 1.05%. After pre-treating cells with HBLS-L and HBLS-C for 12 h, followed by CoCl_2_ exposure, it was observed that cell viability significantly increased compared to cells treated with CoCl_2_ alone (*p* < 0.01) (Figure 1D,E). The maximum difference in cell viability between HBLS-L and HBLS-C concentrations of 312.5 μg/mL and 62.5 μg/mL, respectively. Therefore, HBLS-L and HBLS-C concentrations were set at 300 μg/mL and 60 μg/mL, respectively. At these concentrations, cell viability in the CoCl_2_ group was significantly lower than the control group, while cell viability in the HBLS-L and HBLS-C groups was significantly higher than the CoCl_2_ group (*p* < 0.01) (Figure 1F). Under an inverted microscope, cells in the CoCl_2_ group exhibited shrinkage, rounding, and even detachment from the culture dish, while HBLS-L and HBLS-C treatments reduced these morphological changes (Figure 1G). Measurement of lactate dehydrogenase (LDH) levels in the culture supernatant revealed that LDH levels were significantly higher in the CoCl_2_ group compared to the control group, while LDH levels in the HBLS-L and HBLS-C groups were significantly lower than the CoCl_2_ group (Figure 1H) (*p* < 0.01).

### 3.2. Metabolomics Analysis Based on LC-MS/MS

**Figure 2:**
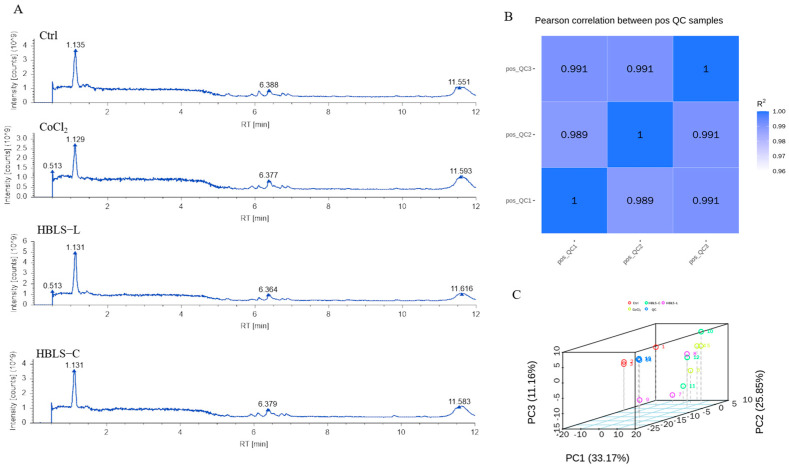
In the positive ion mode, QC analysis was performed on all samples using LC−MS to generate PCA plots. (**A**) Representative total ion chromatograms of each group’s samples. (**B**) Correlation analysis of QC samples. (**C**) PCA of the entire sample set.

The LC-MS/MS total ion chromatogram (TIC) of the Ctrl, CoCl_2_, HBLS-C, and HBLS-L groups is shown in Figure 2A. This instrument aligned the peak retention times and mass deviations for different samples, ensuring more accurate identification and quantification of peak areas, meeting the testing requirements. The stability and data quality of the entire detection process depends on R^2^, where a higher correlation R^2^ value for the QC samples indicates greater stability and data quality. In this study, all R^2^ values were greater than 0.98 (Figure 2B). PCA revealed that the Ctrl group was furthest from the CoCl_2_ group, followed by the HBLS-C group, and closest to the HBLS-L group, indicating that the Ctrl group showed the largest difference from the CoCl_2_ group, while the HBLS-C and HBLS-L groups minimized the differences (Figure 2C).

**Figure 3:**
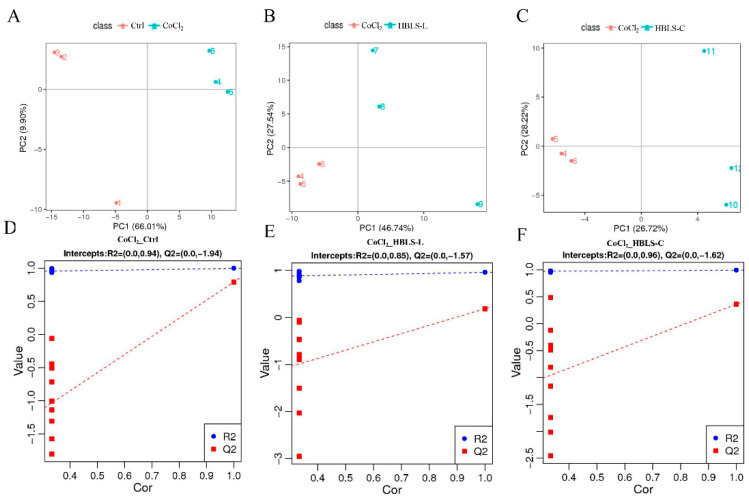
Partial least squares discriminant analysis and validation plots for (**A**,**B**) Ctrl group and CoCl_2_ group. (**C**,**D**) PLS-DA and validation plots for the CoCl_2_ group and HBLS-L group. (**E**,**F**) PLS-DA and validation plots for the CoCl_2_ group and HBLS-C group.

PLS-DA is a supervised discriminant analysis statistical method. After 7 rounds of cross-validation, a model was obtained. It is evident from the figure that the CoCl_2_ group is well separated from the Ctrl, HBLS-L, and HBLS-C groups in the principal components, indicating significant differences (Figure 3A–C). Subsequently, 200 permutations were performed to establish regression lines based on Q^2^ and R^2^. A high-quality and stable model is indicated when the R^2^ value is greater than Q^2^, and the Q^2^ regression line intersects the *Y*-axis at a value less than 0. The results show that the R^2^ and Q^2^ values for the Ctrl vs. CoCl_2_ groups are 0.094 and −1.94, respectively; for the CoCl_2_ vs. HBLS-L groups, they are 0.085 and −1.57, respectively; and for the CoCl_2_ vs. HBLS-C groups, they are 0.96 and −1.62, respectively (Figure 3D–F). Hence, the model meets the required quality standards.

**Figure 4:**
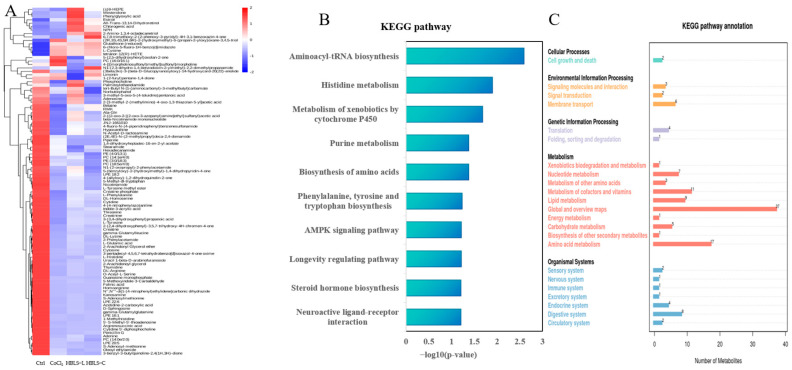
Differential metabolite clustering analysis and KEGG enrichment results. (**A**) Heatmap of differential metabolite clustering in four groups. (**B**) KEGG Pathway. (**C**) KEGG Pathway annotation.

A total of 289 metabolites were identified through differential metabolite analysis. Compared to the control group, the CoCl_2_ group showed upregulation in 5 metabolites and downregulation in 65 metabolites. In comparison to the CoCl_2_ group, the HBLS-L group exhibited upregulation in 14 metabolites and downregulation in 4 metabolites. Additionally, the HBLS-C group showed upregulation in 10 metabolites and downregulation in 5 metabolites (Figure 4A). Further enrichment analysis using the KEGG pathway revealed the major biological functions influenced by the differential metabolites, including Aminoacyl-tRNA biosynthesis, histidine metabolism, metabolism of xenobiotics by cytochrome P450, purine metabolism, biosynthesis of amino acids, phenylalanine, tyrosine and tryptophan biosynthesis, AMPK signaling pathway, longevity regulating pathway, Steroid hormone biosynthesis, and neuroactive ligand-receptor interaction (Figure 4B). Additionally, the KEGG pathway annotation highlighted the impact of differential metabolites on cell growth and death (Figure 4C).

### 3.3. HBLS Extract on the Effects of CoCl_2_-Induced H9C2 Cell ROS Generation, Apoptosis, and MMP

**Figure 5:**
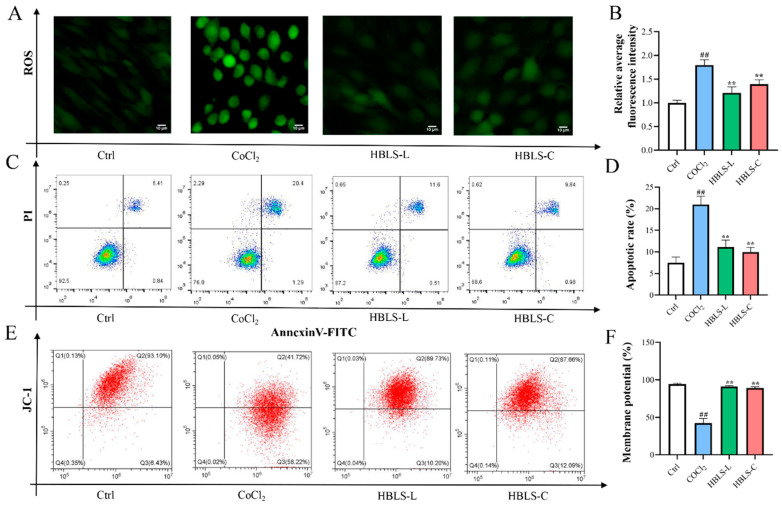
HBLS extract on the effects of CoCl_2_-induced H9C2 cell ROS generation, apoptosis, and MMP. (**A**) Representative fluorescent images of ROS production in cells stained with DCFH-DA. (**B**) Bar graph of relative fluorescence intensity of ROS compared to the Ctrl group. (**C**) Representative scatter plot of apoptosis analyzed by Annexin V-FITC/PI double staining and flow cytometry. (**D**) Cell apoptosis rate. (**E**) Representative scatter plot of MMP analyzed by JC-1 staining and flow cytometry. (**F**) Fluorescence intensity of JC-1 staining. Data are presented as mean ± SD, indicated as ^##^ *p* < 0.01 compared to the Control group, and ** *p* < 0.01 compared to the CoCl_2_ group, respectively.

In order to determine the potential protective effect of HBLS extract against CoCl_2_-induced H9C2 cell damage, we assessed the levels of ROS using fluorescence probe technique and measured cell apoptosis and mitochondrial membrane potential using flow cytometry. The results revealed that the ROS levels in the CoCl_2_ group were significantly higher compared to the Ctrl, HBLS-L, and HBLS-C groups (*p* < 0.01) (Figure 5A,B). The apoptosis rate of H9C2 cells in the CoCl_2_ group was significantly higher than in the other groups (*p* < 0.01) (Figure 5C,D). Furthermore, the mitochondrial membrane potential in the CoCl_2_ group was significantly lower than in the Ctrl, HBLS-L, and HBLS-C groups (*p* < 0.01) (Figure 5E,F).

### 3.4. HBLS Extract Activated Cell Apoptosisthe and AMPK Pathway in CoCl_2_-Induced H9C2 Cells

**Figure 6:**
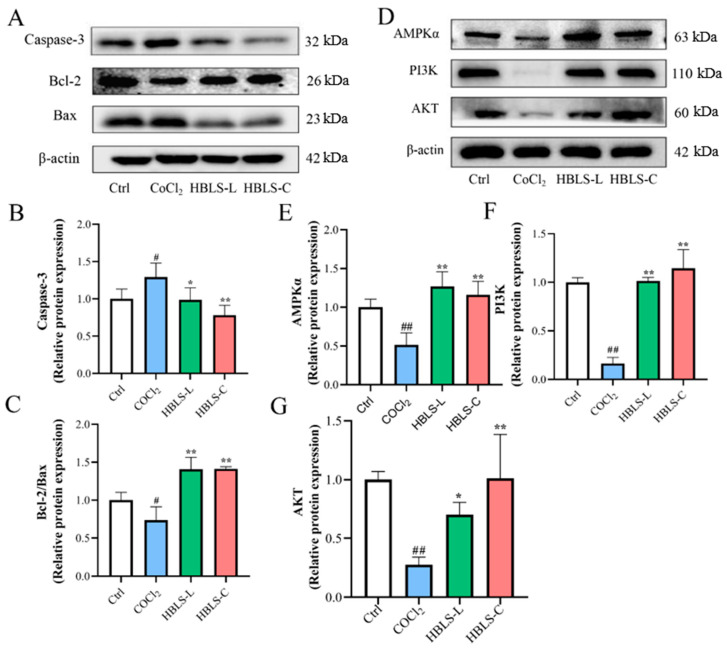
HBLS extract alleviates CoCl_2_-induced apoptosis and damage in the AMPK pathway (see Supplementary Materials). (**A**) Representative protein blot analysis of apoptosis. (**B**) The relative protein expression of Caspase-3. (**C**) The relative protein expression of Bcl-2/Bax. (**D**) Representative protein blot analysis of the AMPK pathway. (**E**) The relative protein expression of AMPKα. (**F**) The relative protein expression of PI3K. (**G**) The relative protein expression of AKT. Data are presented as mean ± SD, indicated as ^#^ *p* < 0.05 and ^##^ *p* < 0.01 compared to the Control group, and * *p* < 0.05 and ** *p* < 0.01 compared to the CoCl_2_ group, respectively.

Based on the Western blot results, it was observed that the level of Caspase-3 in the CoCl2 group was significantly higher than that in the Ctrl group, and the levels of Caspase-3 in the HBLS-L group and HBLS-C group were lower compared to the CoCl2 group. In contrast, the Bcl/Bax level was significantly lower in the CoCl_2_ group compared to the other groups (*p* < 0.05, *p* < 0.01) (Figure 6A–C). The protein concentrations of AMPKα, PI3K, and AKT in the CoCl_2_ group were significantly lower than those in the Ctrl group (*p* < 0.01). However, the protein concentrations of AMPKα, PI3K, and AKT in the HBLS-L group and HBLS-C group were significantly higher than those in the CoCl_2_ group (*p* < 0.05, *p* < 0.01) (Figure 6D–G).

## 4. Discussion

The rapid development of metabolomics provides a new avenue for studying the therapeutic effects of traditional Chinese medicine (TCM) in treating diseases. TCM formulas are diverse in components and rich in action targets. In the treatment of cardiovascular diseases, they can conduct holistic conditioning, regulate multiple aspects such as blood lipids and blood pressure through multiple targets, and also strengthen the healthy qi and eliminate pathogenic factors. Since they are composed of natural ingredients, TCM formulas have fewer side effects and a mild action, which is beneficial for long-term conditioning. However, due to the complex characteristics of TCM formulas, their mechanism of action cannot be explained solely by a single approach to disease treatment [20]. By employing omics-based methods, researchers can investigate the mechanisms of TCM formulas from various perspectives, thus further facilitating their clinical application and development [21,22].

It has been found that the use of herbal medicine alone or in combination can lead to changes in metabolite profiles [23]. Zou et al. utilized metabolomics techniques to analyze metabolite differences and evaluate the effects of the compound Danshen Dripping Pills on myocardial ischemia in rats [24]. In this study, metabolomics techniques were used to screen a total of 289 metabolites, with 70 differential metabolites between the CoCl_2_ group and the control group, 18 differential metabolites between the HBLS-L group and the CoCl_2_ group, and 20 differential metabolites between the HBLS-C group and the CoCl_2_ group. Principal component analysis (PCA) and partial least squares-discriminant analysis (PLS-DA) revealed significant differences between the CoCl_2_ group and the control group, as well as between the HBLS-L group and the CoCl_2_ group, and the HBLS-C group and the CoCl_2_ group. Analysis of KEGG pathways and KEGG pathway annotations unveiled the impact of CoCl_2_ on the AMPK signaling pathway and cellular growth and death processes in H9C2 cells. Therefore, to determine whether the HBLS extract provides protection against damage to H9C2 cells further investigation was conducted.

Cell apoptosis is a programmed cell death process that is closely related to the cell’s lifecycle and function [25]. Multiple studies have indicated that CoCl_2_ induces apoptosis in myocardial cells, and reducing apoptosis is beneficial for myocardial cell protection [26,27]. In this study, it was observed that HBLS-L and HBLS-C increased the survival rate of CoCl_2_-induced H9C2 cells, restored the normal structure of myocardial cells, and reduced LDH activity levels in the culture medium. The number of apoptotic cells, as determined by Annexin-V-FITC/PI flow cytometry, was lower in the HBLS-L and HBLS-C groups compared to the CoCl_2_ group. The production of ROS is directly related to cellular events such as protein oxidation and misfolding. Excessive accumulation of ROS leads to the accumulation of unfolded proteins in the endoplasmic reticulum, further inducing cell apoptosis [28]. This study found that the ROS levels in the CoCl_2_ group were relatively higher than those in the control group, while the HBLS extract groups were lower than the CoCl_2_ group (*p* < 0.01). Furthermore, HBLS extracts reversed the decrease in JC-1 membrane potential in CoCl_2_-induced H9C2 cells. Mitochondrial membrane potential is an important indicator of mitochondrial integrity, and the decrease in mitochondrial membrane potential is considered one of the characteristics of cell apoptosis [29,30]. Therefore, the effect of HBLS extracts on membrane potential can also demonstrate their inhibitory effect on CoCl_2_-induced cell apoptosis. CoCl_2_-induced injury in H9C2 cells regulates other endogenous apoptosis cascades, including Caspase-3, Bcl-2, and Bax [31]. Cellular degenerative changes are mediated by the activation of Caspase-3, which initiates the execution phase of the apoptosis cascade. In addition, Bcl family proteins and other apoptosis indicators interact with Caspase-3 [32]. Bcl-2 and Bax are two important proteins in the Bcl protein family that play multiple roles in apoptosis, with Bcl-2 having anti-apoptotic effects and Bax having pro-apoptotic effects [33,34,35]. In this study, the modulation of Caspase-3, Bcl-2, and Bax by HBLS extracts was observed, indicating that both HBLS-C and HBLS-L can inhibit the apoptosis cascade in CoCl_2_-induced H9C2 cells. These findings suggest that HBLS extracts have a protective effect on CoCl_2_-induced H9C2 cells.

AMPK is an important physiological energy sensor and a key regulator of cellular metabolism. It plays an essential role in controlling glucose intake, glycolysis, as well as protecting the heart from ischemic injury and cell apoptosis [36]. The regulation of cellular metabolism by AMPK is highly associated with heart disease, and the activation of AMPK has been shown to have beneficial effects in preventing ischemic heart damage [37]. Western blot results demonstrated that HBLS extract enhanced the protein expression of AMPK in CoCl_2_-induced H9C2 cells, indicating its protective role against myocardial cell injury. The PI3K/AKT pathway responds to changes in nutrient availability and metabolic products in the body and cells [38]. AMPK can activate the PI3K/AKT pathway, and studies have shown that activation of the PI3K/AKT pathway significantly reduces the expression of Bax protein [39,40]. Additionally, this pathway is involved in regulating ROS homeostasis, promoting cell growth and proliferation [41]. Western blot results showed that CoCl_2_ significantly reduced the protein expression of PI3K and AKT in H9C2 cells, while HBLS extract reversed this phenomenon, suggesting that the protective effect of HBLS extract on H9C2 cell injury is positively correlated with the activation of the AMPK/PI3K/AKT signaling pathway.

## 5. Conclusions

The extraction of HBLS has provided a new research approach aided by metabolomics. Our study results demonstrate the efficacy of HBLS extract in suppressing myocardial cell damage, which is positively correlated with the activation of the AMPK signaling pathway.

## Figures and Tables

**Table 1:** Description of HBLS Powder.

Medicinal Plant	Voucher Specimen Number	Local Name	Ratio	Origin (China)	Key Phytochemical Compounds	Effect	Ref.
Rhodiola rosea (rhizome of *Rhodiola rosea* L.)	NJAU-CVM-2022005	Hong Jingtian	4	Xizang	Salidroside, Kaempferol, Quercetin et al.	The monarch herb increases the cardiac output in heart failure.	[12]
Atractylodes (radix of *Atractylodes macrocephala* Koidz.)	NJAU-CVM-2022006	Bai Zhu	3	Sichuan province	Biatractylenolide II, Luteolin, Isoscopoletin, et al.	The minister herb is widely used in the treatment of coronary artery disease	[13]
Gei Herba (*Geum aleppicum* Jacq.)	NJAU-CVM-2022007	Lan Buzheng	1	Xizang	Flavonoids, Phenylpropanoids, Tannins, et al.	The assistant herb improves peripheral hemogram promotes hematopoiesis	[14]
Codonopsis (radix of *Codonopsis pilosula* (Franch.) Nannf.)	NJAU-CVM-2022008	Dang Shen	2	Xizang	Polysaccharides, Alkaloids, Flavonoids, et al.	The assistant herb improves cardiac function of infarcted hearts	[15]

## Data Availability

All data can be provided as needed.

## Supplementary Materials

The following supporting information can be downloaded at: https://www.mdpi.com/article/10.3390/vetsci12030267/s1.
