# Supplementary figures and images for: Hong-Bai-Lan-Shen Extract Alleviates the CoCl2-Induced Apoptosis in H9C2 Cells by Regulating the AMPK Pathway
Source: Vet Sci. 2025 Mar 13;12(3):267. doi: 10.3390/vetsci12030267 (PMC11946019; doi:10.3390/vetsci12030267)

### Caspase-3

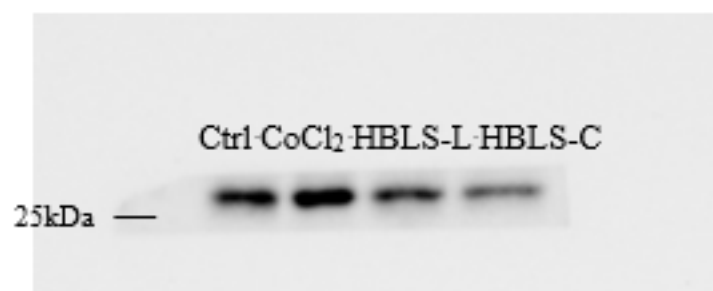

### Bcl-2

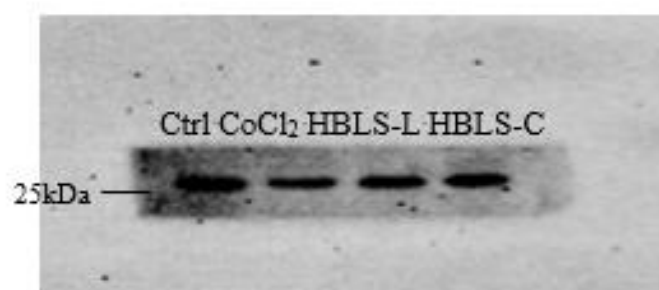

### Bax

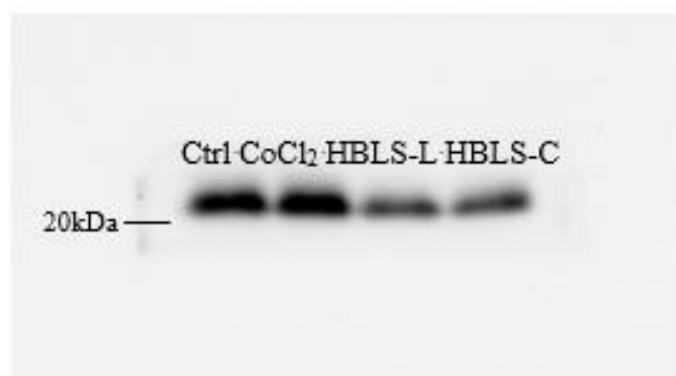

AMPK $\alpha$

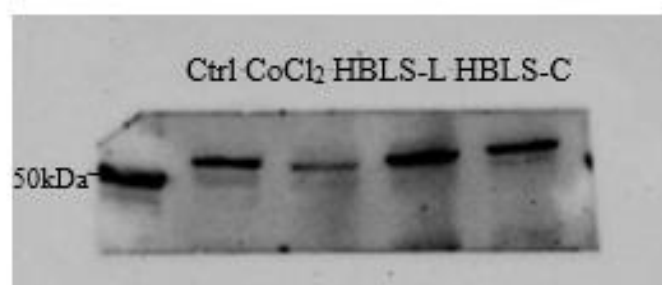

PI3K

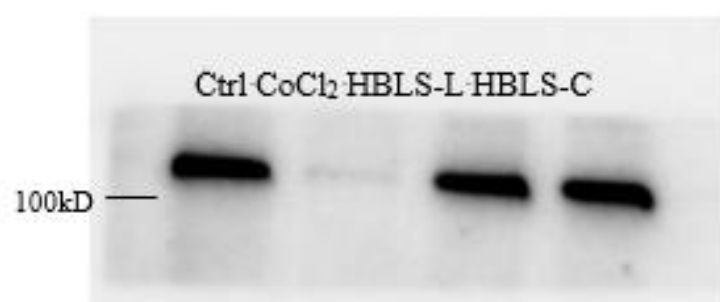

AKT

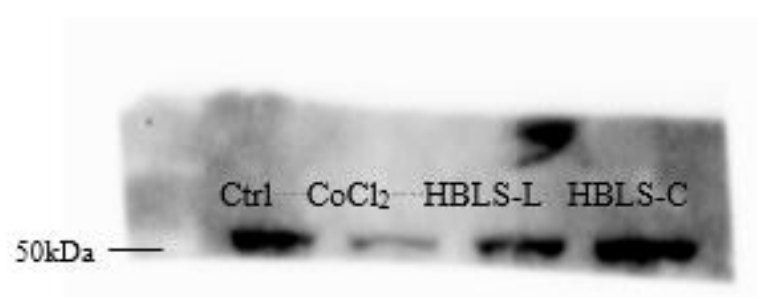

Supplement: Supplementary file 1 [file vetsci-12-00267-s001.zip › Original WB -D.pdf]

$\beta$ -actin

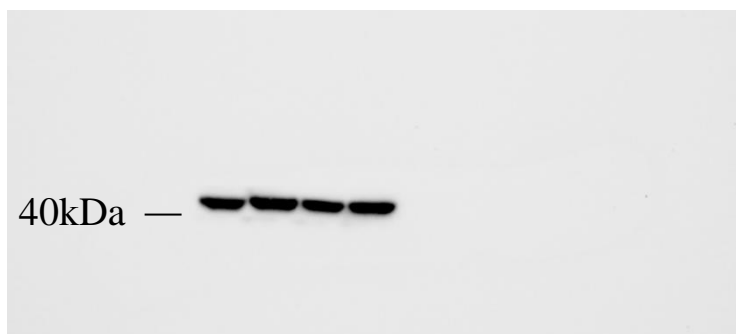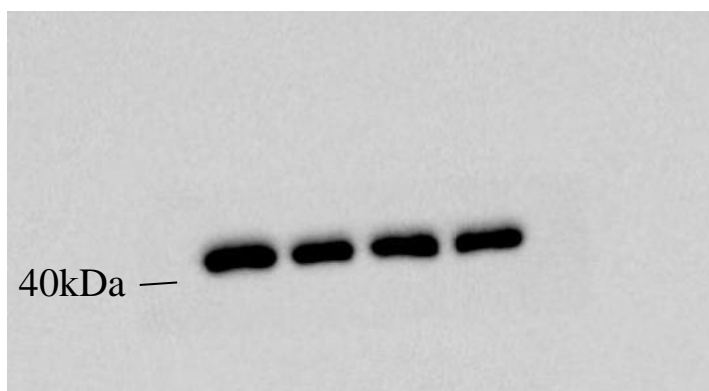

Supplement: Supplementary file 1 [file vetsci-12-00267-s001.zip › a┬-actin.pdf]
